# Recycled Glass Fiber Composites from Wind Turbine Waste for 3D Printing Feedstock: Effects of Fiber Content and Interface on Mechanical Performance

**DOI:** 10.3390/ma12233929

**Published:** 2019-11-27

**Authors:** Amirmohammad Rahimizadeh, Jordan Kalman, Rodolphe Henri, Kazem Fayazbakhsh, Larry Lessard

**Affiliations:** 1 Department of Mechanical Engineering, McGill University, Montreal, QC H3A0C3, Canada; amirmohammad.rahimizadeh@mail.mcgill.ca (A.R.); larry.lessard@mcgill.ca (L.L.); 2 Department of Aerospace Engineering, Ryerson University, Toronto, ON M5B2K3, Canada; jkalman@ryerson.ca; 3 Institute of Materials (IMX), Ecole Polytechnique Fédérale de Lausanne (EPFL), CH-1015 Lausanne, Switzerland; rodolphe.henri@alumni.epfl.ch

**Keywords:** wind turbine blades, polylactic acid, glass fiber, fused filament fabrication, 3D printing

## Abstract

This research validates the viability of a recycling and reusing process for end-of-life glass fiber reinforced wind turbine blades. Short glass fibers from scrap turbine blades are reclaimed and mixed with polylactic acid (PLA) through a double extrusion process to produce composite feedstock with recycled glass fibers for fused filament fabrication (FFF) 3D printing. Reinforced filaments with different fiber contents, as high as 25% by weight, are extruded and used to 3D print tensile specimens per ASTM D638-14. For 25 wt% reinforcement, the samples showed up to 74% increase in specific stiffness compared to pure PLA samples, while there was a reduction of 42% and 65% in specific tensile strength and failure strain, respectively. To capture the level of impregnation of the non-pyrolyzed recycled fibers and PLA, samples made from reinforced filaments with virgin and recycled fibers are fabricated and assessed in terms of mechanical properties and interface. For the composite specimens out of reinforced PLA with recycled glass fibers, it was found that the specific modulus and tensile strength are respectively 18% and 19% higher than those of samples reinforced with virgin glass fibers. The cause for this observation is mainly attributed to the fact that the surface of recycled fibers is partially covered with epoxy particles, a phenomenon that allows for favorable interactions between the molecules of PLA and epoxy, thus improving the interface bonding between the fibers and PLA.

## 1. Introduction

The extensive disposal of composite products is leading to undesirable environmental impacts due to their high organic content such as resin and wood [1,2]. Recent reports have revealed that the total global production of composites has exceeded 10 million tonnes per year, which, at the end of life, will require over 5 million cubic meters for disposal [3]. Among composite materials, the superior mechanical properties of glass fiber reinforced polymers (GFRP) combined with low cost have made them an attractive alternative for solid materials, resulting in 90% use in all of composites currently produced [4]. Aircraft, automotive parts, pipes, and sports equipment are some examples of application sectors for GFRPs [5,6]. Among GFRP products, wind turbine rotor blades serve as one of the major application sectors of GFRPs, which has undergone a significant growth since the year 2000 [7,8,9]. A wind turbine blade generally consists of two glass fiber composite shells, which are adhesively bonded together. In addition, they typically contain a core material, e.g., balsa wood or foam, which is used in some sections of the blade with the aim of reducing the production cost. The accelerating growth of wind energy, which translates to a production of 6 million tonnes of GFRP over the coming decade, has highlighted the issue of composite recycling. Although current disposal methods, such as incineration or landfill offer simple and low-cost solutions for the blade waste disposal, the rapidly increasing cost, imminent recycling legislation, as well as the lower availability of landfill sites, have made such disposal techniques economically and socially unacceptable [10,11,12,13]. Many countries are now increasing landfill taxes to steer the industry into finding new solutions [14]. Nevertheless, several thermal and chemical techniques are offered in the literature for the recycling of thermoset GFRPs [2,15]. However, these methods are generally associated with a substantial loss in the mechanical properties of the recovered fibers as compared to virgin first-pass materials [16,17]. In addition, their inability to handle large quantities of waste highly limits their applicability.

Currently, mechanical grinding is considered as the only recycling technique with commercialization potential [18]. This method was first introduced in the 1990s and involves the breakdown of composites by shredding, milling, grinding, or other size reduction processes. The aim of this technique is to reduce the size of the scrap composites and reincorporate them as reinforcement or filler into new composite structures. Since mechanical grinding is not associated with any atmospheric pollution, this technique offers significant environmental and economical advantages over the other available recycling processes. In addition, this process benefits from the abrasive nature of glass fibers to efficiently reduce the size of GFRP composite wastes [19]. The performance and functionality of this method has been the focus of a significant number of studies in the literature [20,21]. For instance, Palmer et al. [18,19] used a TRIA screen-classifier type hammer mill to recover glass fibers from automotive front fenders. They validated the applicability of a novel zig-zag air separator method to obtain fine glass fibers featuring mechanical properties comparable to virgin fibers. In another study, three grades of granulated recyclate from sheet moulding compound waste with different fiber sizes were obtained and used in a bulk moulding compound (BMC) manufacturing to replace the virgin reinforcements [22]. The new BMC formulations showed reduced mechanical properties, particularly in flexural strength. This observation was ascribed to possible fiber damage, and poor interfacial bonding between the fibers and the new resin polymer.

While mechanical grinding has been known as an effective and efficient recycling technique, it is strictly highlighted in the literature that the viability of this method relies on the careful separation of characterized fibers with length comparable to virgin fibers [18]. As a result, short fiber reinforced composites could be deemed as a superior potential application for mechanically recycled composites. One of the recent application sectors for short fiber composite materials, which is growing fast, is fused filament fabrication (FFF) 3D printing. Benefiting from a layer upon layer manufacturing technique, FFF provides an exciting opportunity for rapid fabrication of parts with complex geometry [23,24,25]. Recently, a new class of 3D printing filaments integrating reinforcement materials, namely, short and continuous fibers, with pure thermoplastic polymers to improve their mechanical performance, has been introduced [26,27,28,29,30]. Zhong et al. [27] exploited the effect of short glass fibers on the mechanical properties of acrylonitrile butadiene styrene (ABS) fabricated parts. They presented composite filaments with three distinct fiber contents, which could enhance the surface rigidity and tensile strength of the traditional pure ABS filaments. Papon et al. [31] showed that the addition of short carbon fibers into neat PLA can improve the fracture properties of 3D printed parts. In another study, continuous carbon fiber bundles were used to reinforce 3D printed polycarbonate parts. The results from this study demonstrated a substantial increase of, respectively, 77% and 85% in the tensile yield strength and modulus of elasticity of parts printed with three fiber bundles [30]. The latest advances in the AM technology include efforts to improve the sustainability of this widespread manufacturing process via the use of recycled materials. For instance, an optimized fused particle fabrication method has been recently developed, which allows for reusing recycled polymers to manufacture 3D printed parts with properties comparable to those of virgin components [32,33]. More recently, Rahimizadeh et al. [34] validated a recycling process to reclaim short glass fibers from scrap rotor blades for use in 3D printing. The results from this study showed that the stiffness and strength of the reinforced filament are improved by 16% and 8%, respectively. In addition, an improvement of 8% was reported for the stiffness of the composite samples made of recovered fibers. While interesting, in this study, the effect of the resin residue on the interface bonding developed between the recycled fibers and the new resin system, as well as the influence of fiber content on the properties of the recycled composites were not studied.

The aim of the current investigation is twofold: (i) to experimentally examine the effect of the recycled fibers extracted from rotor blades wastes on the tensile properties of FFF fabricated specimens. Filaments with varying fiber content are extruded and used to fabricate tensile test specimens as per ASTM D638-14. The effect of the fiber content on the mechanical properties and porosity of the FFF 3D printed samples is evaluated (ii) to investigate the impregnation of the recycled fibers and PLA. 3D printing filaments containing recycled glass fibers and virgin glass fibers with silane coating are produced and used to fabricate tensile test specimens. Fracture interface of these is then observed using scanning electron microscopy (SEM) to analyze the interfacial strength between the fibers and resin. At the end, the closing remarks and routes for future studies are discussed.

## 2. Materials and Methods

In Section 2.1, we first explain the recycling and manufacturing process developed to obtain recycled glass fiber reinforced filament. The steps include mechanical recycling, pelletizing and filament extrusion. Section 2.2 details the fabrication process of additively manufactured recycled tensile test specimens as per ASTM D638.

### 2.1. Recycling and Extrusion Procedure

**Figure 1:**
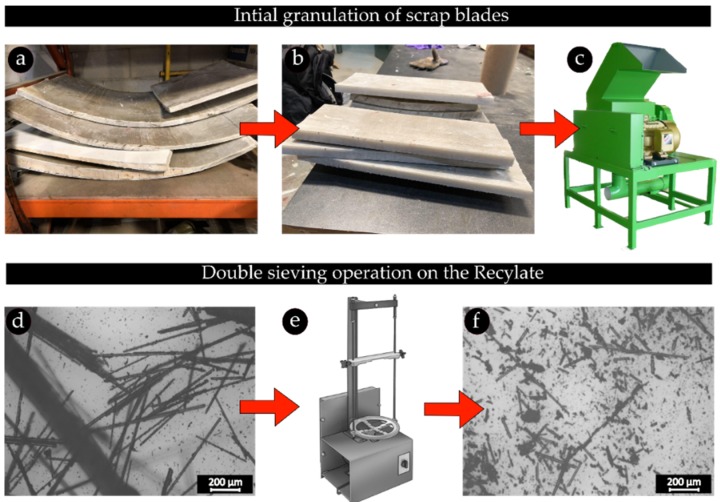
The recycling process to obtain glass fibers processable for FFF 3D printing: (**a**) scrap wind turbine blades; (**b**) 20 × 20 cm pieces of the scrap blades; (**c**) hammer mill grinder used to grind the pieces; (**d**) ground recyclate obtained after the initial granulation of the blade pieces; (**e**) double sieving operation on the ground recycled materials; (**f**) the last grade of the recyclate obtained after the second sieve operation.

Figure 1 shows the recycling scheme, which is used to extract the glass fibers from wind turbine blades. Mechanical grinding of the end of life turbine blades is used to extract the reinforcement fibers from the blades. In this process, the scrap blades are first cut into 20 × 20 cm pieces and fed into a hammer mill grinder (ECO-WOLF, INC., Edgewater, FL, USA) equipped with a 3 mm classifier screen. As can be observed in Figure 1b, only sections made of pure fiberglass composites are used in this study. As a result, the recyclate compound obtained after the grinding process is not isolated glass fibers, rather a mixture of glass fibers and particles of resin.

Since the presence of fibers longer than the 3D printer nozzle diameter can lead to nozzle clogging and jamming, we resort to a double sieving operation to separate off the fibers with length above 0.4 mm. As a result, the recycled materials are classified through an 8” ASTM stainless-steel sieve with a mesh size of 140. Following this process, the sieved material is further classified through another sieving operation to obtain better refined fibers in the desirable length range. Although efficient and simple, the sieving mechanism is generally associated with the possibility of contamination with longer fibers as the high aspect ratio fibers might fall through the sieve meshes.

**Figure 2:**
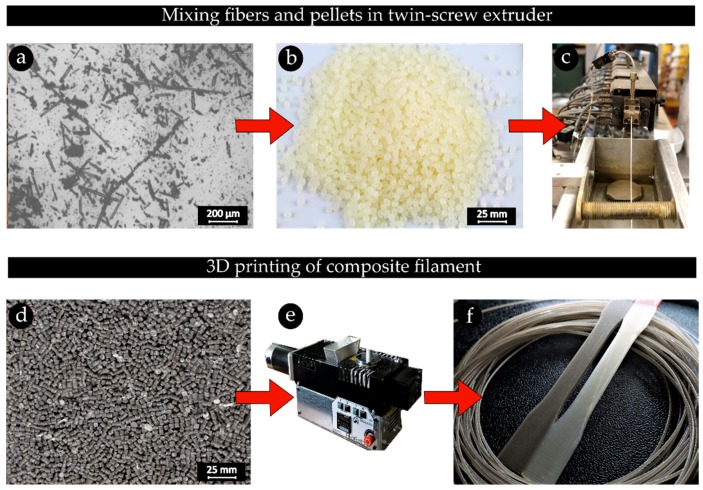
The double extrusion process to obtain 3D printing composite filament: (**a**) the last grade of the recyclate; (**b**) PLA pellets; (**c**) palettization process with a twin-screw extruder; (**d**) the glass fiber reinforced pellets; (**e**) single screw filament extruder; (**f**) the recycled glass fiber reinforced filaments and composite tensile test specimens.

To characterize the recycled materials, in particular the epoxy residue content, thermogravimetric analysis (TGA) is performed. TGA is conducted in order to assess the amount of retained matrix residue inside the recyclate. Samples weighing 7 mg are extracted from the initial and last grade of the recyclate and loaded into a TGA System (Q500, TA Instruments, New Castle, DE, USA). A heating program in the range of 20–800 °C with a heating rate of 10 °C/min is then used to decompose the resin powder. The TGA is performed in the presence of nitrogen followed by an oxidative stage such that no ash content is left on the surface of the fibers. One crucial factor contributing to the performance and processability of short fiber reinforced 3D printing filaments is the fiber distribution. To homogeneously distribute the fibers within PLA, as shown in Figure 2, we resort to a double extrusion process where the recycled materials are first mixed with virgin PLA pellets (Ingeo 4043D, Natureworks LLC, Blair, NE, USA) and fed into a twin-screw extruder (Leistritz ZSE18HP-40D, Nuremberg, Germany) with 8 subzones. Using a pelletizer, the produced composite filament is then chopped up into small pellets featuring different fiber contents including 5 wt%, 10 wt%, 15 wt%, 20 wt% and 25 wt%. It should be recalled that the reclaimed recyclate materials with no prior pyrolysis are directly used in this pelletization process.

Subsequently, the composite pellets are fed into a single screw extruder (FilaFab, D3D Innovations Limited, Bristol, UK) to obtain composite filaments with different fiber contents. Using an air-cooling system, the filament is cooled down immediately after the extrusion and its diameter is consistently monitored through a laser micrometer with ±2 µm accuracy. To minimize the possible diameter variations throughout the filament, a spool winder machine is utilized and the extrusion parameters including the die temperature, speed of the winder, as well as the screw speed are well adjusted to attain a 1.75 ± 0.05 mm filament. This range of variation represents an acceptable tolerance and diameter for the FFF process. The temperature profiles used in the extrusion processes are obtained through a trial and error method on the standard parameters recommended by the material supplier. These parameters are optimized to produce filaments with acceptable surface quality and no degradation in the polymer [34]. Table 1 shows all the process parameters used during the first and the second extrusion.

### 2.2. Characterization of Material Properties

Tensile test samples in accordance with the ASTM D638 Type I specimen geometry and dimensions are manufactured and used to evaluate the mechanical performance of the recycled composite parts. Samples with a total thickness of 3.36 mm and a stacking sequence of [0]_24_ are manufactured using a Prusa i3 Mk2S printer (Prusa Research, Prague, Czech Republic). Seven specimens for each fiber content (0 wt% or pure PLA, 5 wt%, 10 wt%, 15 wt%, 20 wt% and 25 wt%) are manufactured, for a total of 42 specimens. Then, the samples are weighed using a precision balance with an accuracy of 0.01 g. Design and manufacturing parameters for the FFF process are reported in Table 2. These parameters were optimized in a research study to manufacture specimens with high surface quality and dimensional accuracy [35]. A 313Q tensile machine from Testresources with a 50 kN load cell is used for tensile testing at a 5 mm/min displacement rate. The fracture interface of the specimens is evaluated using scanning electron microscopy (Hitachi UHR Cold-Emission FE-SEM SU8000, Hitachi, Ltd, Chiyoda, Tokyo, Japan). To examine the role of fiber surface coating on the impregnation level of the recycled fibers and PLA and compare to that of virgin glass fibers, representative virgin fiber reinforced filaments with 10 wt% fiber content are produced and used to manufacture test specimens. The stacking sequence and thickness of these samples are identical to those of the recycled glass fiber specimens. The virgin glass fibers (MEF-11-100, Shenzhen Yataida High-Tech. Co., Ltd., Shenzhen, China) are coated with silane sizing and feature an average diameter of 0.017 mm and a fiber length of 0.007–0.4 mm, dimensional properties similar to those of recycled fibers. Therefore, the interface developed between the two different composite systems, namely recycled fibers/PLA and virgin fibers/PLA, can be surmised to contribute to the relative mechanical performance of theses composites.

## 3. Results

This section presents results for the proposed mechanical recycling scheme, which is used to fabricate and evaluate FFF samples out of recycled composite filament and pure PLA filament, a commercially available baseline. In addition, the interfacial bonding between the recycled fibers and PLA is evaluated using the approach described in Section 2.2.

### 3.1. Mechanical Grinding

**Figure 3:**
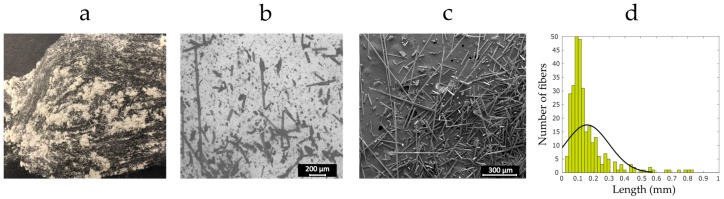
Recycled fibers: (**a**) the final grade recyclate; (**b**) an optical microscopy image from the recycled compound; (**c**) a SEM micrograph from the recycled compound; and (**d**) probability distribution for fiber length of the last grade of ground fibers.

The recycling system presented in Section 2.1 resulted in the recyclate compound shown in Figure 3a. In order to characterize the length of the fibers, a group of at least 300 fibers from the last grade of the sieved recycled compound was sized. Figure 3d plots the distribution of data versus the fiber length. The yellow rectangles show the experimental measurements, and the solid line is the Gaussian curve of the best fit. As seen, most of the fibers feature a length of below 0.4 mm, a critical characteristic to the processability of the fiber reinforced filaments for the FFF 3D printing process. The average fiber length is around 0.19 mm. Although fibers with length of above 0.4 mm are visible in the recycled materials, the final grade of the recyclate appears to have suitable fiber length distribution. In addition, the double sieving operation has yielded recyclate compound consisting of individual refined fibers with no fiber bundles.

**Figure 4:**
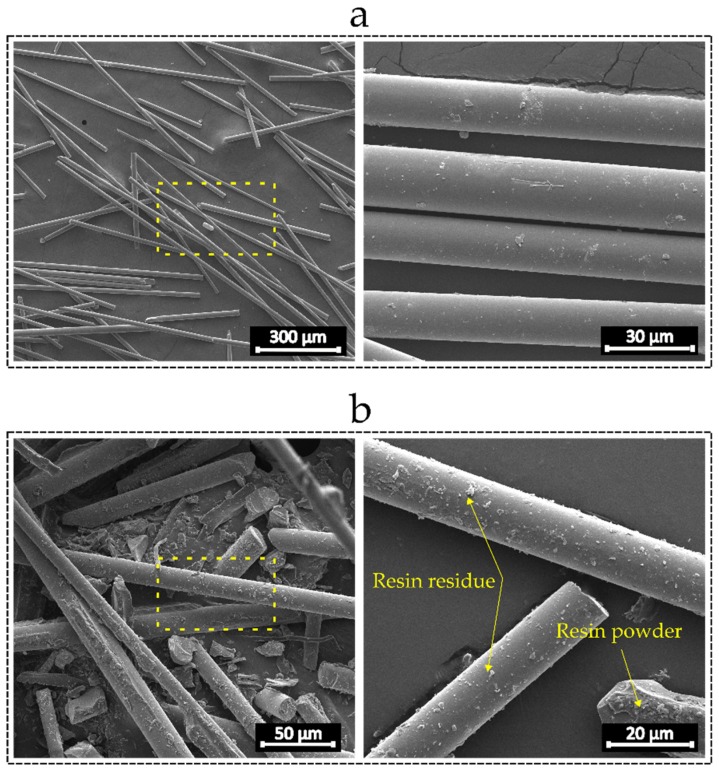
SEM micrographs: (**a**) virgin glass fibers; and (**b**) recycled glass fibers.

Figure 4 illustrates the structural morphology of the recycled and virgin glass fibers. The SEM micrographs show that the recycled fibers retained a significant amount of epoxy residue on the surface, while the virgin fibers have a thin and uniform silane coating layer. Powdered epoxy particles are also visible in the recycled materials. On one hand, the epoxy residue powder decreases the surface quality of the fibers, which could lead to local stress concentration and thus lower strength. On the other hand, it could increase the fiber surface roughness enabling mechanical interactions between the PLA molecules and the fibers. In addition, it is demonstrated in the literature that epoxy has the potential to form hydrogen bonding with PLA, which could also further enhance the impregnation level of the recycled fibers and PLA [36].

**Figure 5:**
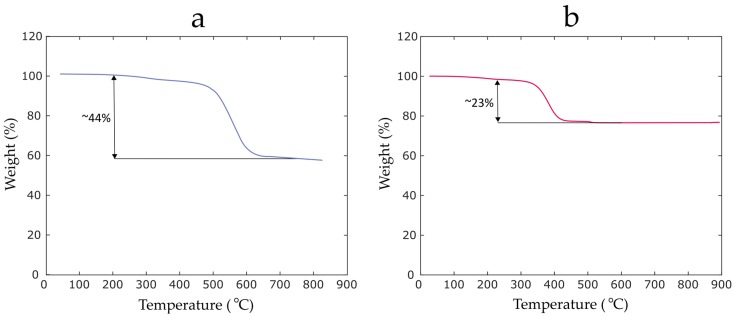
The thermogravimetry thermograms of the recyclate materials: (**a**) ground recyclate; (**b**) last grade of the recyclate obtained after the second sieve operation.

An important factor in defining the added value of recycled materials is the resulting enhancement in mechanical performance when used in a new material system. This occurs when the maximum amount of reinforcing glass fibers is preserved during the sieving of the ground recycled materials. The level of the reclaimed fibers is evaluated here by performing TGA on the recyclate obtained after the initial granulation and the second sieving mechanism. As it can be observed in Figure 5, while the ground recycled materials approximately contain 44% of the resin residue, the second sieving operation enabled a proper separation of the powder particles such that almost 77% of the fibers are retained. This indicates that the last grade of the recyclate can provide a better reinforcement as compared to the initial ground materials.

### 3.2. Recycled Composite 3D Printing Filaments

**Figure 6:**
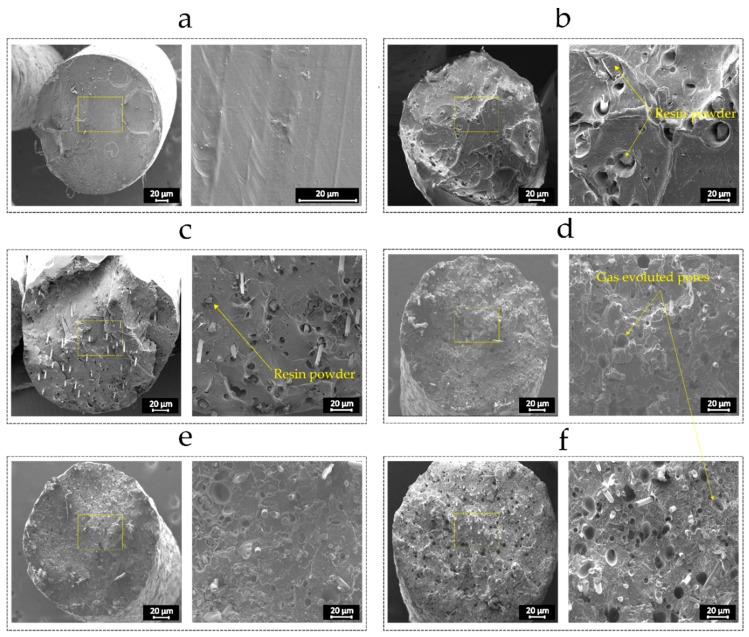
Microstructures of FFF 3D printing filament with different fiber content: (**a**) 0 wt%; (**b**) 5 wt%; (**c**) 10 wt%; (**d**) 15 wt%; (**e**) 20 wt%; and (**f**) 25 wt%.

The microstructure of the pure PLA filament and the reinforced ones with different recycled fiber contents is displayed in Figure 6. As seen, the pure PLA filament features a solid cross section with no visible voids. However, the addition of the recycled glass fibers leads to visible porosities in the filament, which get increasingly larger as the fiber content increases. In addition, non-impregnated resin particles with sharp corners appear in the composite filaments. Apart from potential stress concentration regions, relatively small pores are formed around these particles, which could affect the properties by generating the possibility of micro crack formation.

### 3.3. Mechanical Characterization of the FFF 3D Printed Tensile Specimens

**Figure 7:**
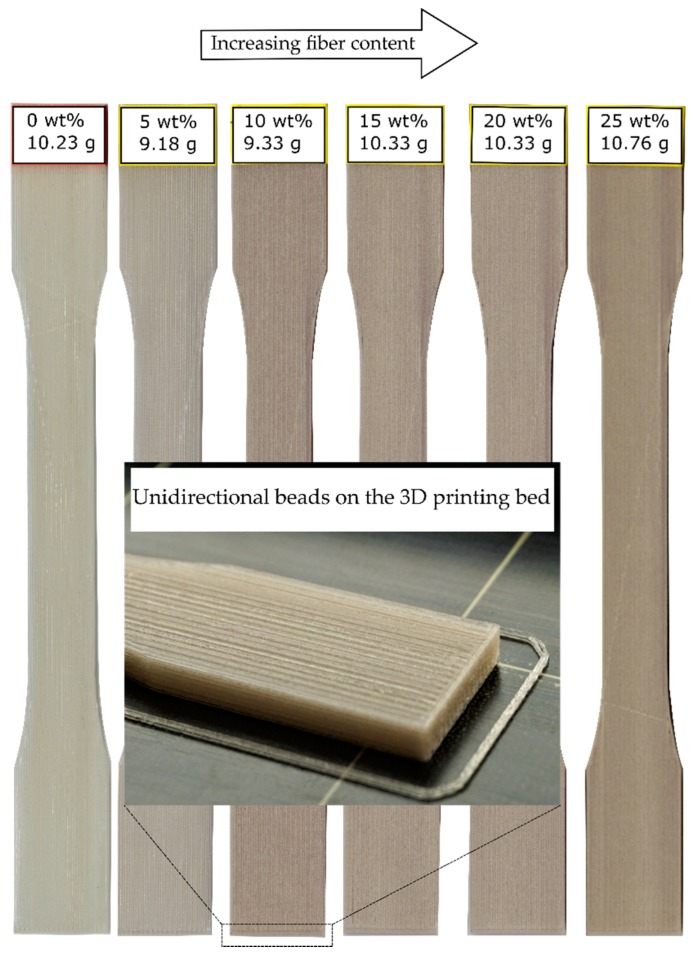
The FFF 3D printed coupons with unidirectional bead orientation and different fiber content.

**Figure 8:**
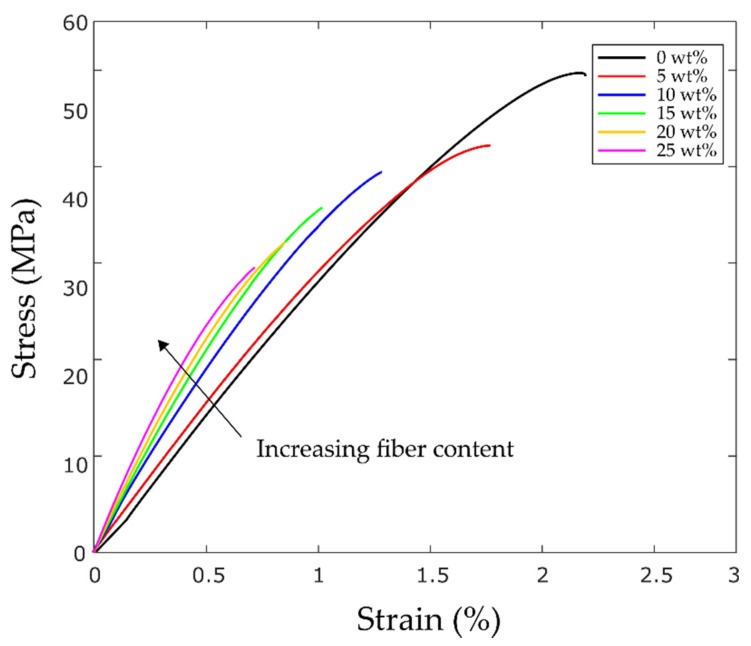
Stress–strain curves of pure PLA and recycled glass fiber reinforced specimens.

Figure 7 shows one 3D printed specimen per each fiber content. The unidirectional bead orientation can be seen in all samples. A comparison of the typical stress vs. strain curves of the specimens is shown in Figure 8. The curves are selected from the results of seven specimens.

**Figure 9:**
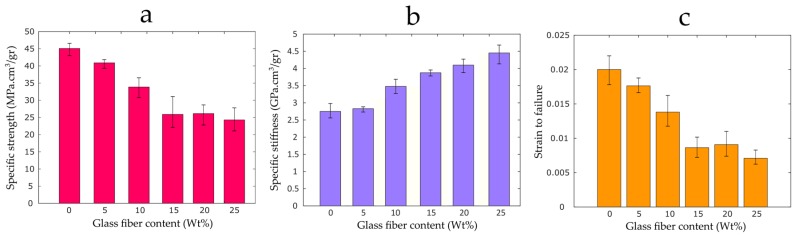
Tensile properties of glass fiber reinforced specimens with different fiber content: (**a**) specific stiffness; (**b**) specific strength; and (**c**) failure strain.

Figure 9a,b illustrate the specific tensile strength and stiffness of the 3D printed coupons. Due to some minor variations in the weight of the samples, the normalized specific properties are reported. The results indicate that, as the fiber content increases, the mean specific tensile stiffness of the samples improves, leading to structurally stiff components. A significant increase in specific tensile modulus occurs from 5 wt% to 10 wt%. The maximum specific stiffness is observed for the 25 wt% coupons with an improvement of 74% (2.56 GP.cm^3^/gr to 4.45 GP.cm^3^/gr). The error bars in Figure 9 show the standard deviation (SD) of multiple tests. SD values can be mainly ascribed to the variation of the fiber length, resin powder, as well as the discrepancy between the fibers surface roughness, which arises from the epoxy particles remained on the surface of the ground fibers. As per the specific strength, lower values are measured for the composite samples. The results show that, as the fiber content increases, the specific tensile strength of the samples reduces, such that a reduction of 42% is observed for the 25 wt% coupons.

As shown in Figure 9c, the ductility of the 3D printed samples also reduces with the increase of the fiber content. The pure PLA samples show the largest failure strain of 2%. On the other hand, the composite samples with 25 wt% recycled fibers lead to the smallest mean failure strain of about 0.7%, which suggests a reduction of about 65%.

**Figure 10:**
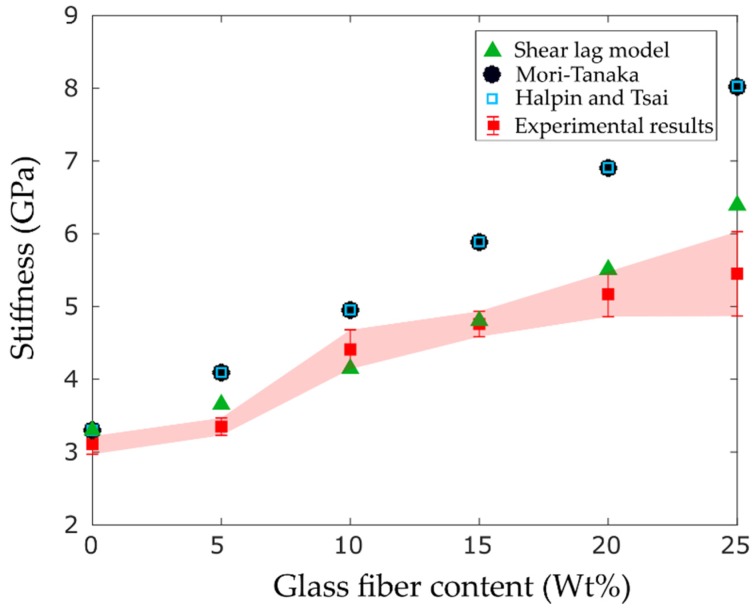
Experimental and analytical stiffness results of short fiber composites versus fiber content.

Different established analytical models including Mori–Tanaka, shear lag, and Halpin–Tsai are used to predict and compare the elastic stiffness of short glass fiber composites to that of the 3D printed parts [37,38]. The theories are briefly described in Table 3. In these equations, $E_{f}$, $E_{m}$, v, and l/d denote the fiber stiffness, matrix stiffness, fiber volume fraction, and fiber aspect ratio, respectively. In addition, S, $C_{c}$, $C_{m},$ and $C_{i}$ represent the Eshebly tensor, and stiffness tensor of the composite, matrix, and fiber, respectively. As displayed in Figure 10, the theoretical models showed a similar trend as the experimental results and they agree well, particularly at low weight fractions. However, the high viscosity of the polymer at high weight fractions coupled with nozzle clogging increased the porosity of the 3D printed parts leading to larger difference between the experimental results and the analytical models.

**Figure 11:**
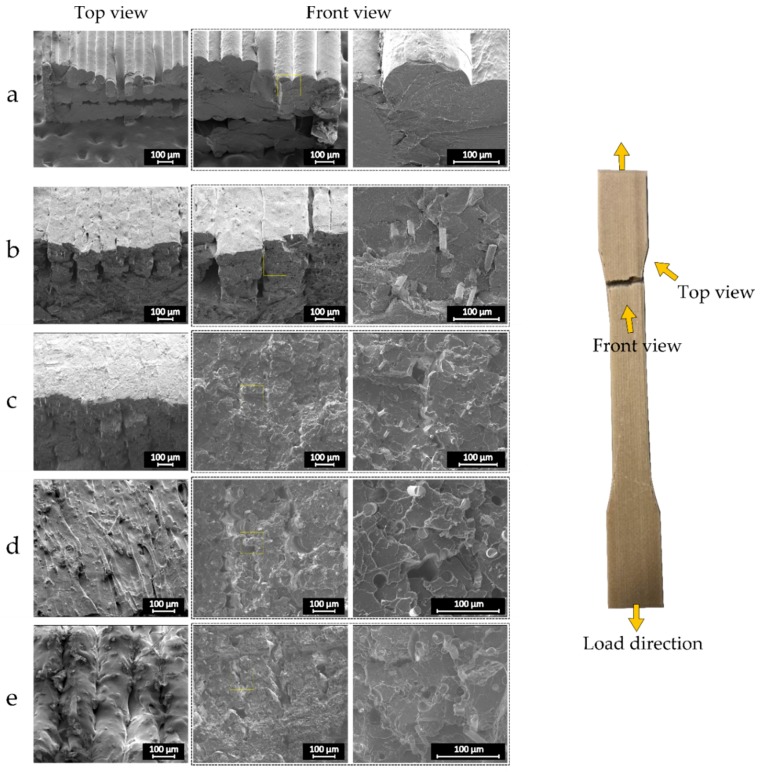
Representative SEM micrographs of the composite samples with (**a**) 0 wt%; (**b**) 10 wt%; (**c**) 15 wt%; (**d**) 20 wt%; (**e**) 25 wt% fiber content.

To further investigate the reasons behind the mechanical behavior of the composite 3D printed specimens, their microstructure at the fracture surface is studied. Figure 11 shows the scanning electron micrographs of representative samples from the top and front views. As can be seen, the addition of the recycled fibers substantially reduced the surface quality of the coupons. It was observed that the surface quality of the coupons is consistent with the fiber content. The lower the fiber content, the better surface quality. As the fiber content of the filament increases, the distribution of the fibers within the beads aggravates such that some of the fibers are not deposited properly and are not embedded within PLA. This phenomenon generates local stress concentration regions, which could be one reason for the low strength of the composite samples. Another cause for the lower strength of the composite samples could be the presence of resin particles and fibers with ineffective length in the critical zones, which boost the possibility of microcrack formation. The nonuniform distribution of resin particles on the surface of fibers, particularly at fiber ends, could be another culprit for the reduction in ultimate strength of the composite samples. Moreover, the composite samples with higher fiber content contained a higher quantity of pores. This observation could be ascribed to the presence of large pores in the composite filaments and also the incidence of nozzle clogging when printing with higher fiber content filaments. Previous works showed a similarity in the strength and stiffness trend for 3D printed composite parts [39,40]. For example, in a recent study on hemp hurd/PLA filament, while an increase in the volume fraction of the fiber improved the part stiffness, it was associated with a larger quantity of voids, which led to lower tensile and flexural strength for the 3D printed samples [41]. In another study, 3D printed graphene-based ABS parts featured lower tensile strength and ductility as compared to virgin ABS samples [42]. Two main possible reasons for this property degradation were identified as high viscosity of the polymer, as well as the local stress concentration induced by graphene nanoplates. This observation confirms the stress raiser effect of the epoxy particles dispersed in the recycled glass fiber composite parts.

### 3.4. Interfacial Bonding Strength

**Figure 12:**
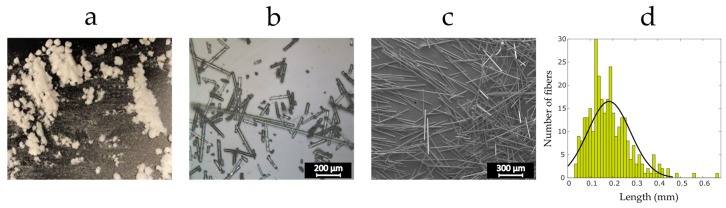
Virgin fibers: (**a**) the reinforcement fiber compound; (**b**) an optical microscopy image from the virgin fibers; (**c**) a SEM micrograph from the virgin fibers; and (**d**) probability distribution for the fiber length of the virgin fibers.

To examine the bonding strength at the interface between the PLA and the ground fibers, a relative study is performed between tensile test specimens reinforced with virgin glass fibers and samples made out of recycled fibers. To do so, representative samples with 10 wt% virgin and recycled glass fibers are manufactured and tested. Figure 12b,c show, respectively, a microscopic and a SEM image of the virgin fibers, illustrating isolated glass fibers with varying length. As seen, the length distribution of the virgin fibers is similar to that of recycled fibers. Furthermore, they feature an average fiber length of approximately 0.19 mm, a dimensional characteristic identical to that of recycled fibers. This ensures that any difference in the samples properties is only due to the interfacial strength between the fibers and the resin.

**Figure 13:**
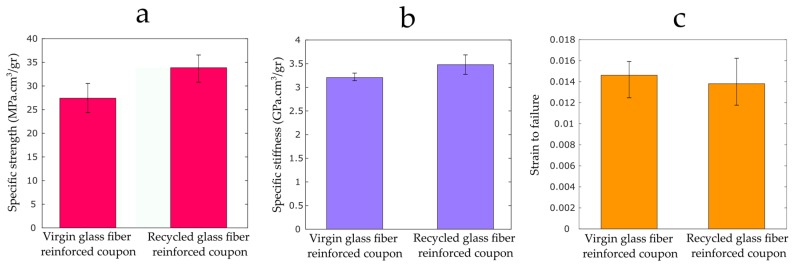
Tensile properties of specimens reinforced with recycled and virgin fiber glass with 10 wt% fiber content: (**a**) the specific strength; (**b**) the specific stiffness; and (**c**) strain to failure.

The mechanical properties of the samples with both the virgin and recycled fibers are shown in Figure 13. Noticeably, the samples with recycled fiber glass showed higher specific strength and stiffness, with values 19% and 8% higher than those of specimens reinforced with virgin fibers. However, slightly higher failure strain with value as high as 5% was observed for the virgin samples. This value of failure strain pertains to the lower stiffness of the virgin samples.

**Figure 14:**
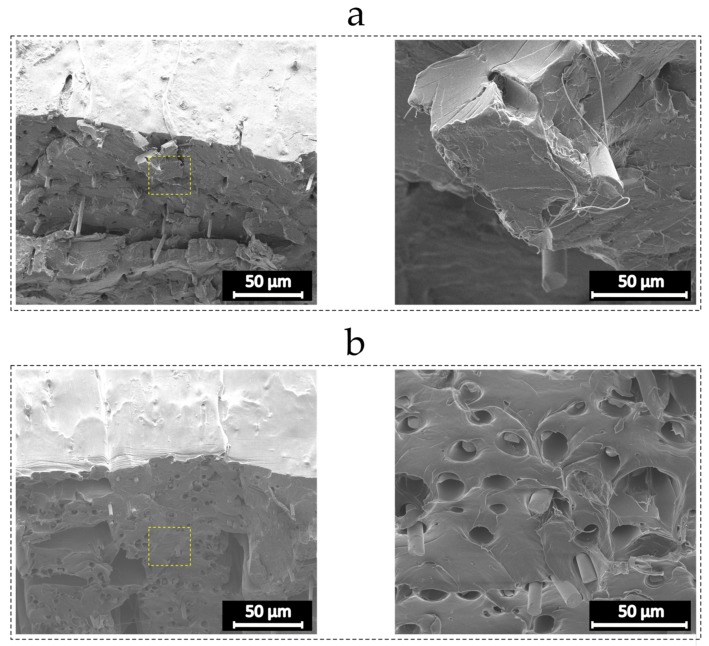
SEM micrographs from the fracture surface of tensile specimens: (**a**) recycled glass fiber reinforced specimen; and (**b**) virgin glass fiber reinforced specimen.

As shown in Figure 14, the SEM images from the fracture surface of the samples with virgin and recycled fibers reveal a better impregnation of the recycled fibers and PLA as compared to virgin glass fibers. Although pulled out fibers can be detected on the fracture surface of the recycled samples, most of the recycled fibers are broken and embedded within PLA. On the other hand, many holes on the fracture surface of the specimen reinforced with virgin fibers appear, which show fully pulled out fibers. The presence of epoxy residues on the surface of fibers can contribute to the interfacial strength by providing the possibility of mechanical and chemical interactions between PLA and fibers, where the former results from fiber surface roughness and the latter arises from hydrogen bonding [36]. This indicates an excellent opportunity to fabricate structurally stiff and light weight components with the use of ground fibers from scrap rotor blades.

Previous studies reported structural performance of 3D printed samples reinforced with virgin fibers [43,44]. Mechanical properties obtained for PLA specimens reinforced with recycled glass fibers in this study can be compared with values from the literature. As shown in Table 4, the strength and stiffness of 3D printed specimens in the current study are, respectively, 14% and 108% higher than those of virgin carbon fiber reinforced ABS parts with identical fiber content. On the other hand, for virgin carbon fiber reinforced PLA, the mean value of the strength and stiffness are, respectively, 38% and 18% higher than those of recycled glass fiber reinforced PLA. Besides using virgin fibers, this discrepancy arises from higher mechanical properties of carbon fibers with respect to recycled glass fibers. Nevertheless, given the low price of PLA and free access to composite wastes from rotor blades, the results of this work offer the opportunity to replace the existing composite filaments with an inexpensive material featuring comparable properties.

### 3.5. Economic and Environmental Advancement

Interest in recycling of composites is rapidly increasing as the composite material industry strives to become more environmentally friendly. It is significant to be at the forefront of recycling technology to reduce the carbon footprint of composites. Considering the limited lifespan of wind turbine blades, which is between 20–25 years, the first generation of blades will be decommissioned in the near future. Disposing of these blades will be a major issue as it is not feasible to merely cut up the blades and put them in a landfill. According to economic analysis, the landfilling cost for a material flow of 10,000 Mg/Y of turbine blades is about 837 K USD, which is the most economical feasible solution, presently available [45]. However, the environmental legislations in many countries restrict the landfill of materials with more than 10% organic materials, including turbine rotor blades.

**Figure 15:**
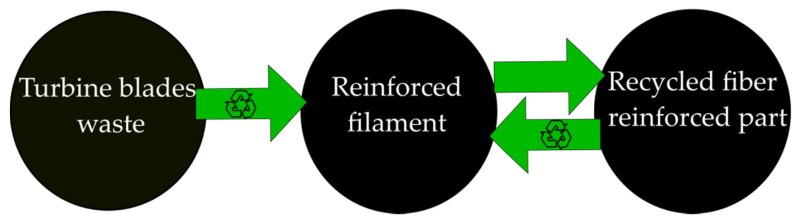
The sustainability pattern for the recycled fiber reinforced composites.

The main contribution of this work relies on a sustainable cost-effective way using an efficient grinder to convert the processed fiberglass into manageable short fibers. This approach will avoid the need for high-energy conversion processes, such as pyrolysis, for recycling. A recent study on the recycling of wind turbine blades has highlighted that the primary energy requirement for mechanical, thermal, and chemical recycling of glass fiber thermoplastic reinforced blades are, respectively, 0.29 MJ/Kg, 1 MJ/Kg, and 4 MJ/Kg, values which indicate the high efficiency of the grinding process for glass fiber composites [3]. One major obstacle hindering the use of mechanical recycling is the loss of high aspect ratio fibers. That being said, the results of this investigation have shown that short fiberglass-reinforced polymers can lead to stiffer 3D printed components with added value for the end users. Using a sustainable and biodegradable polymer, the proposed process will be sustainable since the 3D printed parts made by fiberglass reinforced polymer filaments could also be reused and re-cycled back into the same system, making the entire process waste free, as shown in Figure 15. One critical factor contributing to the applicability of the proposed recycling scheme is the length of the fibers in the recycled compound. Weight statistics investigation shows that approximately 55–60% of the ground recyclate comes out of the sieve after the second sieve operation and the remaining recycled material is not processable. However, the non-sieved residue could be either further ground and reused in the same recycling system or processed using 3D printers with larger nozzle diameters.

As a result, the proposed methodology is associated with significant environmental benefits by providing a closed-looped recycling system for wastes from wind turbine blades. Despite the low cost of virgin glass fibers, which in fact demonstrate the increasing utilization of GFRPs, the viable and economical applications for recycled waste composites will become more profitable in the near future.

The results of this study can serve as the foundation for further work to accurately investigate the entire life cycle of the proposed material. For instance, a study on the recycling and remanufacturing of the 3D printed parts is required. In addition, the mechanical behavior, e.g., strength, of the 3D printed parts should be further studied to bring their performance up in the levels of advanced applications. Other polymers, like ABS, can also be considered to explore their interaction and adhesion to recycled fibers. In addition, other types of fibers, e.g., carbon from other end-of-life products can be the subject of future studies.

## 4. Conclusions

The focus of this work is on the recycling of end-of-life wind turbine blades and reusing them in a sustainable 3D printing process. It was shown that the recovered short glass fibers from scrap turbine blades can be effective in enhancing the structural stiffness of FFF 3D printed components. An innovative and economically efficient recycling scheme integrating mechanical grinding and double sieving mechanism was employed to obtain glass fibers from scrap rotor blades. The SEM images from the recycled fibers showed traces of epoxy on the surface of the fibers. Through tensile experiments on 3D printed specimens reinforced with recycled glass fibers, our results have shown that the use of recycled fibers can substantially improve the structural specific modulus of the components. In contrast, the addition of the fibers had an adverse impact on the ductility and ultimate strength of the samples. The cause for this phenomenon was identified through SEM analysis as the development of excessive stress concentration regions due to two main reasons: first, the fiber aggregates, particularly at high fiber weight fractions and, second, resin particles with sharp corners remained on the surface of the recycled fibers. A comparative study between specimens out of PLA reinforced with virgin glass fibers and recycled fibers showed that the specific strength and stiffness of the samples made of recycled fibers are higher than those of virgin glass fiber reinforced specimens. The SEM micrographs from the fracture surface of these specimens demonstrated a better impregnation of the ground fibers and PLA as compared to virgin fibers. The reason behind this observation is mainly due to the retention of epoxy particles on the surface of the fibers, which offers the opportunity of potential mechanical and chemical interactions between the molecular network of PLA and epoxy. The mechanical interactions can lead to molecular interlocking and the chemical interactions can form hydrogen bonding between PLA and epoxy resulting in chain extension and further enhancement in the interfacial strength. While the recycling methodology here presented was only applied to glass fiber composite waste from wind energy industry, it can be systematically designed and applied to other glass fiber waste resources, e.g., pipes and boat hulls. This will provide the opportunity to take further steps to meeting the demands of recycling glass fiber reinforced plastics from the composite industry.

## Figures and Tables

**Table 1:** The extrusion parameters for the single-screw and twin-screw extruders.

**Initial Extrusion**	Screw speed (rpm)	90
	Subzone 1–2 (Temp °C)	190
	Subzone 3 (Temp °C)	185
	Subzone 4 (Temp °C)	180
	Subzone 5 (Temp °C)	175
	Subzone 6–8 (Temp °C)	170
**Second Extrusion**	Screw speed (rpm)	25
	Die temperature (Temp °C)	210
	Winder speed (rpm)	1

**Table 2:** Manufacturing and design parameters for specimen 3D printing.

Manufacturing Parameter	Value	Manufacturing Parameter	Value
Print direction	XYZ	Nozzle diameter (mm)	0.4
Raster angle	0	Nozzle temperature (°C)	215
Layer height (mm)	0.14	Cooling	No fan cooling
Bed temperature (°C)	60	Infill (%)	100
Print speed (mm/min)	2400	Filament diameter (mm)	1.75

**Table 3:** Analytical models for short fiber composites.

Theory	Expression
Shear lag model	$E_{c}=\lambda E_{f}v+E_{f}\left(1-v\right)$ $\lambda =1-\frac{tanh\eta l/2}{\eta l/2}$ $\eta =\sqrt{\frac{2G_{m}}{E_{f}r_{f}{}^{2}ln\left(\frac{R}{r_{f}}\right)}}$
Mori–Tanaka	$C_{c}=\langle \left(C_{i}-C_{m}\right)B_{i}\rangle {\left[\left(1-v\right)I+v\langle B_{i}\rangle \right]}^{-1}+C_{m}$ $B_{i}={\left[S{\left(C_{m}\right)}^{-1}\left(C_{i}-C_{m}\right)+I\right]}^{-1}$
Halpin–Tsai	$E_{c}=\frac{1+2\left(l/d\right)\eta_{L}v}{1-\eta_{L}v}\;E_{m}$ $\eta_{L}=\frac{\frac{E_{f}}{E_{m}}-1}{\frac{E_{f}}{E_{m}}+2\left(l/d\right)}$

**Table 4:** Comparison of the mechanical properties for the recycled glass fiber reinforced parts and conventional reinforced 3D printed parts.

Authors	Material	Fiber Content (%)	Strength (MPa)	Stiffness (GPa)
Ivey et al. [43]	Carbon fiber/PLA	15	55.2	5.68
Ning et al. [44]	Carbon fiber/ABS	15	35	2.3
Present work	Glass fiber/PLA	15	40	4.8
